# Personalized drug stratification using endoscopic samples to assess ex vivo gastric cancer tissue susceptibility to chemotherapy and immune checkpoint inhibitors

**DOI:** 10.1007/s10238-025-01694-z

**Published:** 2025-06-04

**Authors:** Laura Hennig, Astrid Monecke, Ngoc Anh Hoang, René Thieme, Sebastian Prill, Albrecht Hoffmeister, Jan Tuennemann, Ingo Bechmann, Florian Lordick, Sonja Kallendrusch

**Affiliations:** 1 Institute of Anatomy, University of Leipzig, Leipzig, Germany; 2 Division of Oncology, Department of Medicine, University of Leipzig Medical Center, Comprehensive Cancer Center Central Germany, Leipzig, Germany; 3 Institute of Pathology, University of Leipzig, Leipzig, Germany; 4 Department of Visceral, Transplant, Thoracic and Vascular Surgery, University of Leipzig Medical Center, Leipzig, Germany; 5 Division of Gastroenterology, Department of Medicine, University of Leipzig Medical Center, Leipzig, Germany; 6 Department of Medicine, Institute of Clinical Research and Systems Medicine, Health and Medical University Potsdam, Potsdam, Germany

**Keywords:** Personalized medicine, Tissue culture, Cancer, Susceptibility testing, Patient derived tissue culture

## Abstract

Customizing drug treatments based on individual tumor susceptibility and patient-specific factors is a complex task, highlighting the need for better predictive models tailored to each patient. Preserving tumor architecture is one strategy to address the intricate interactions between stromal cells and tumor cell populations within a patient. In this study, we explored the feasibility of using pretherapeutic endoscopic tissue cultures from patients (ePDTCs) with gastric and esophagogastric junction cancers to assess individual drug susceptibility. We treated these endoscopic tissue slice cultures with 5-FU (1 µM), a modified FLOT regimen comprising 5-FU (10 µM), leucovorin (10 µM), oxaliplatin (20 µM), and docetaxel (0.1 µM), the active metabolite of irinotecan (SN38, at 1 µM and 10 µM) and the PD-1 inhibitor nivolumab (3 µg/ml). Analysis of the tumor tissue revealed stable adaptations to the culture environment, which were further enhanced by adding 2% autologous human serum to the culture media. Dose-dependent responses were observed with SN38 across all samples and individual susceptibility at low concentrations. Both 5-FU and the FLOT regimen as well as PD-1 inhibition, tested at bioavailable dosages, further demonstrated individualized responses in ePDTCs. This study shows that ePDTCs can effectively assess tissue susceptibility to drugs, warranting further investigation in larger cohorts to validate this model’s potential alongside clinical treatments.

**Supplementary Information:**

The online version contains supplementary material available at 10.1007/s10238-025-01694-z.

## Introduction

Gastric and esophageal cancers are major global health challenges, with gastric cancer ranking as the third and esophageal cancer as the sixth leading cause of cancer-related deaths worldwide, collectively accounting for around 1.3 million deaths annually [1]. Due to a lack of early symptoms, these cancers are often diagnosed at advanced stages, with endoscopic sampling and histological assessment forming the foundation of diagnosis. Histological classifications for gastric and esophagogastric adenocarcinomas are diverse, reflecting significant phenotypic heterogeneity [2, 3]. Additionally, gastric cancer is classified based on various molecular criteria. The Cancer Genome Atlas (TCGA) classification is widely recognized, categorizing tumors into chromosomal instable (CIN), microsatellite instable (MSI), EBV-positive, and genomically stable types, further distinguished by levels of aneuploidy and chromosomal instability [4, 5]. TP53 mutations and epithelial-to-mesenchymal transition (EMT) are also influential in classification schemes [6]. However, a universal standard for classification remains absent in clinical practice and research, highlighting both the complexity of these cancers and the pressing need for personalized treatment approaches.

Clinical management primarily depends on tumor stage, molecular features—such as human epidermal growth factor receptor 2 (HER2), programmed cell death ligand 1 (PD-L1), and MSI status—along with patient factors like performance status, age, medical history, and prior treatments. For resectable and advanced metastatic gastric cancers, gastrectomy combined with perioperative chemotherapy remains superior to supportive care alone [7–11]. While perioperative chemotherapy improves overall and progression-free survival, the prognosis remains poor, with median overall survival at 50 months [7]. Distant relapse remains a key reason for post-surgical progression, underscoring the need for personalized approaches, as reliable biomarkers are lacking and individual treatment responses remain unpredictable [12–16].

A system capable of predicting individual treatment response before drug administration could enable treatment tailored to each patient’s drug susceptibility. Given the frequency of severe side effects with chemotherapy and immunotherapy [11, 17, 18], testing therapies on patient-specific tumor samples could guide patient-specific drug selection. Although various ex vivo models exist to predict drug responses, broad clinical adoption has faced challenges, especially since many models do not adequately mimic the tumor microenvironment (TME)—an essential factor in tumor resistance and novel immune therapies [19]. In this study, we present patient-derived tissue slice cultures (PDTC) from endoscopic samples (ePDTC) of gastric and esophagogastric junction adenocarcinoma, evaluating their response to current therapies and assessing their potential for a personalized, clinically applicable approach.

## Results

### Drug testing requires four endoscopic samples to effectively address tumor heterogeneity and process-related dropouts.

**Fig. 1:**
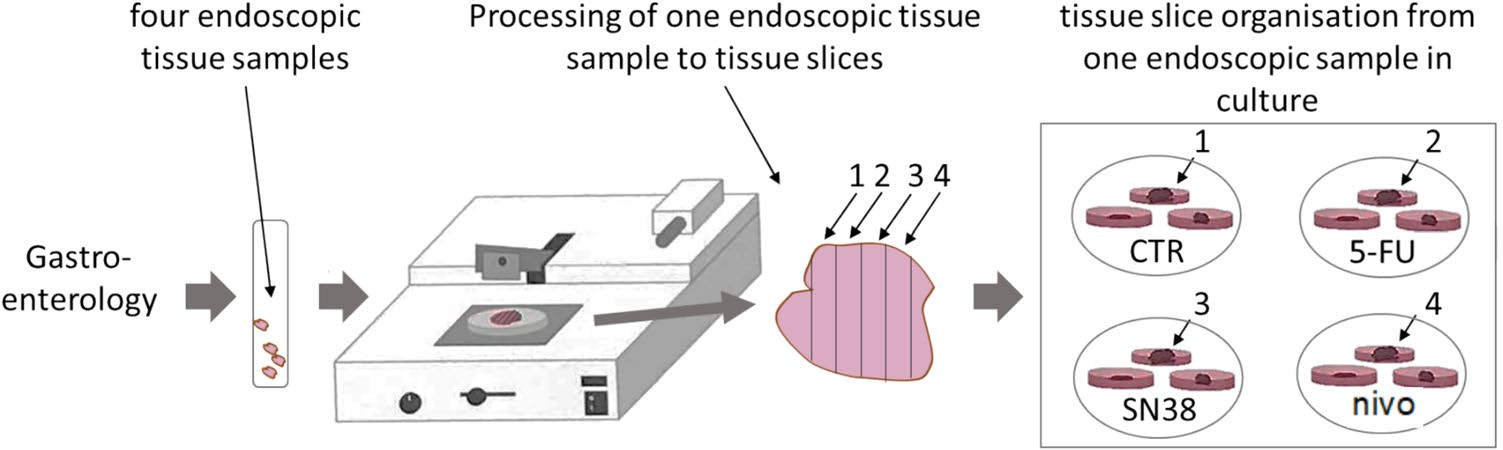
Schematic drawing from clinical biopsy to cultivation. After patient consent is obtained, the gastroenterologist takes samples. Four endoscopic samples are required for susceptibility testing of two to three substances. Each endoscopic sample will be cut into tissue slices, and these single slices need to be distributed to the culture conditions as shown above. As some endoscopic samples drop-out due to quantity or quality, four endoscopic tissue samples are recommended

Endoscopically obtained specimens, ranging from one to seven tissue samples per patient, were investigated and are shown in suppl. Table 1. Depending on the endoscopic tissue sample, four to twelve slices could be obtained from one biopsy. Random selection of the tissue slices, independent of the endoscopic tissue sample to the treatment conditions, was not feasible, as each endoscopic tissue sample yielded a high variation of a specific cellular composition. An equal distribution throughout the culture conditions is necessary to obtain reproducible results. An overview of the method is shown in Fig. 1.

**Fig. 2:**
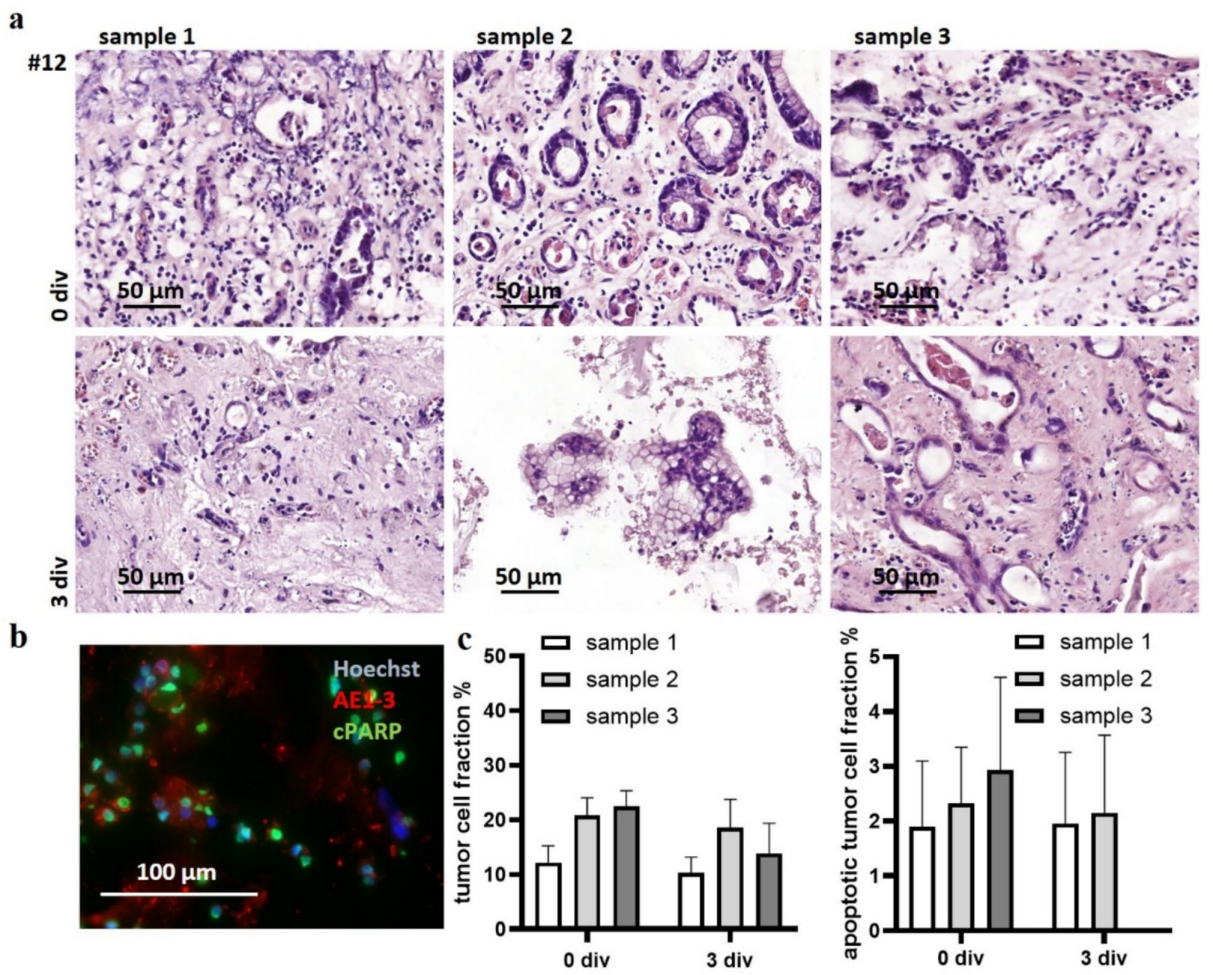
Tissue heterogeneity and rates of apoptosis within three endoscopic samples from one patient. (**a)** Slices from three samples of one signet-ring-cell gastric cancer (#12) were stained with H&E at baseline (0 days in vitro (div)) and after culture in control conditions (3 div). (**b**) Immunostaining for pancytokeratin (AE1-3) and cPARP and counterstaining with Hoechst to calculate the apoptotic tumor cell fraction. (**c**) Shown are the results for each sample from baseline and from the control condition after 3 div. To distinguish the impact of heterogeneity, a duplicate of each condition from every sample was created. No apoptotic tumor cells were visible in the third biopsy. The mean and standard error of the mean (SEM) are shown

Several tumor samples were needed to determine the appropriate tissue quantity and tissue stratification. Biopsies from four patients did not contain any tumor cells and tissue handling required some practice, as crushing of the tissue at any time prior cultivation is detrimental. Several patient samples were needed to determine the right size and right number of biopsies, as some independent conditions developed infections. However, no tissue out of all 31 patient samples investigated dropped-out in total due to infections. First, we determined the tissue heterogeneity between biopsy samples. Three different endoscopic samples from one signet-ring-cell carcinoma are shown in Fig. 2a, representing overall observed biopsy variation. Hematoxylin and eosin staining (H&E) performed directly after preparation showed tumor heterogeneity and fluctuating tumor cell percentages. Different biopsy samples of one patient display all histological variations, e.g., signet ring cells and different amounts of glandular structures. Tissue architecture changes also in some tissue specimen after three days; therefore, we determined tissue response in comparison with the control condition. The control condition is, however, not necessary for determining superiority of one drug, when testing several substances. Immunofluorescent staining of pancytokeratin (AE1-3) and cleaved Poly (ADP-ribose) polymerase (cPARP), counterstained with Hoechst was conducted to address tumor vitality (Fig. 2b). Additionally, the tumor cell fraction and apoptotic tumor cell fraction (Fig. 2c) were measured for each sample, revealing up to a two-fold variation between samples (tumor cell fraction, day 0: 12.18% sample 1; 20.87% sample 2; 22.54% sample 3; apoptotic tumor cell fraction, day 0: 1.90% sample 1; 2.30% sample 2; 2.93% sample 3). After 3 days of cultivation, the tumor cell fraction was well preserved (tumor cell fraction, day 3: 10.30% sample 1; 18.58% sample 2; 13.90% sample 3), and the apoptotic tumor cell fractions also did not show significant alterations compared to baseline samples of day 0 (apoptotic tumor cell fraction, day 3: 1.96% sample 1; 2.15% sample 2). The high variance, inter-sample and intra-sample (Supp. Figure 1) of the same patient in tumor cellularity, proliferation and apoptosis, demonstrate the importance of stratifying the tissue slices to each condition systematically. As some single dropouts, due to tissue adaptations or single slice infections (presumably carried already by the donor), can occur, it is suggested that four endoscopic samples of each individual tumor should be taken to reliably obtain three to four conditions for drug testing.

#### Tumor analysis and pathological examination

**Fig. 3:**
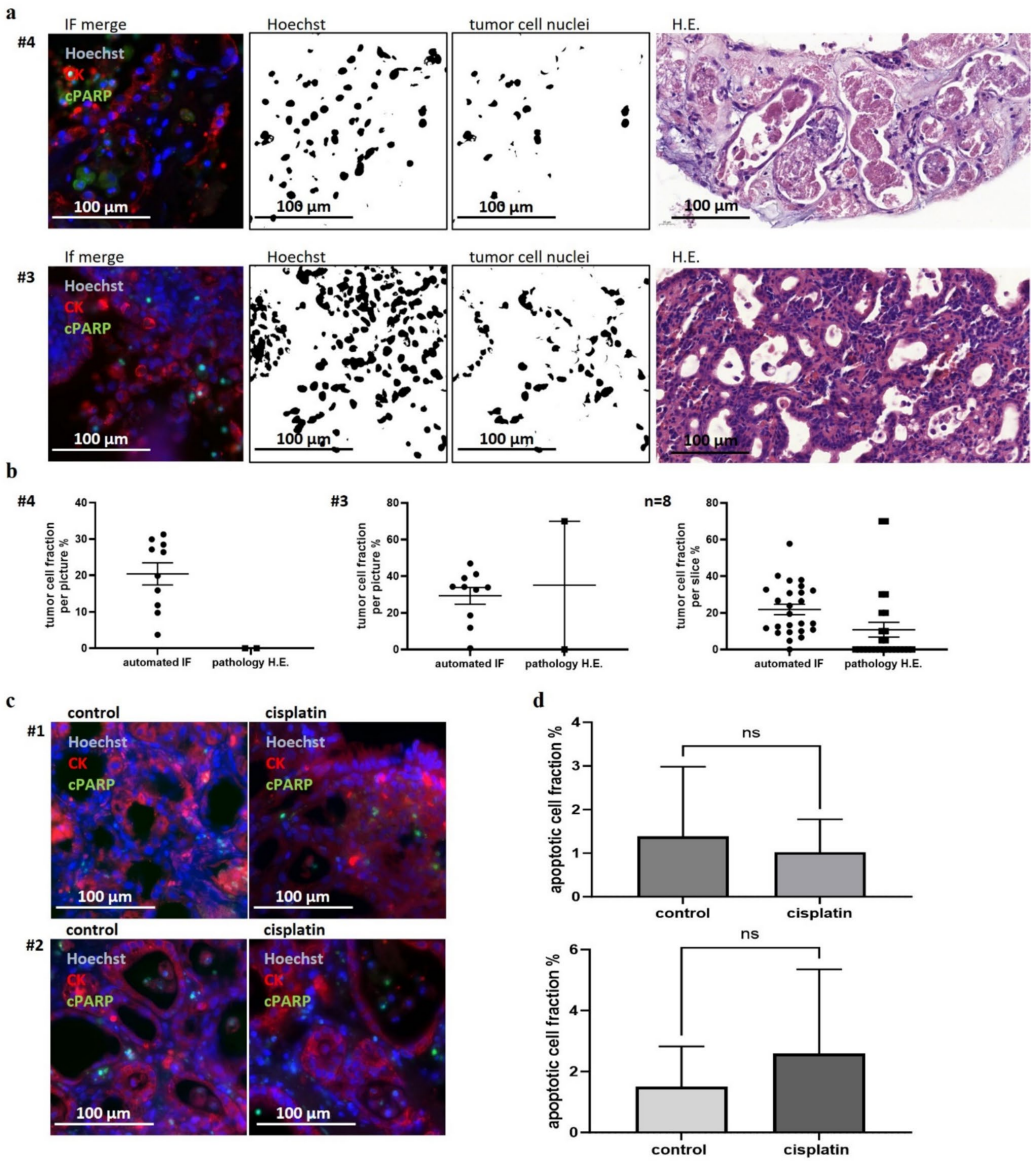
A comparison between automated evaluation of immunofluorescence staining and pathologist evaluation of H&E staining. (**a)** Two representative pictures illustrate the process of automatic tumor cell detection: first, all cell nuclei are identified based on Hoechst staining; in the second step, the nuclei within the area defined by pancytokeratin staining are recognized as tumor cell nuclei. The corresponding H&E stained sections, evaluated by a pathologist, are shown. (**b)** Graphs display the tumor cell fraction for the two experiments, evaluated by a pathologist as well as automated analyzed, shown as the mean with standard error of the mean. Discrepancies are observed in the evaluation for #4, while evaluations converge for #3. The final graph presents a comparison between automatically counted and pathologist counted tumor cell fraction across eight experiments. The mean and the SEM are shown. (**c)** Adjacent tissue from two patients (#1, #2). (**d)** The treated conditions of adjacent tissue (#1, #2) only show a rise in apoptosis in the connective tissues but not in the epithelium. This is not reflected in the analysis, as only cells are considered that express AE1-3. One-way-ANOVA was calculated, and the mean and the SEM are shown

H&E stainings were evaluated for detectable tumor cells in ePDTC by an experienced gastrointestinal pathologist (AM). Pathological analysis showed in eleven out of twelve investigated samples tumor cells (Fig. 3a, b). Analysis of adjacent samples, not containing tumor cells, did display pancytokeratin expression but no regulation of apoptosis after treatment within the epithelial cell population (Fig. 3c, d). Obvious tumor cell aggregations were observed in only few endoscopic tissue slices. Although an overestimation of the tumor cell fraction might occur in single samples, the pancytokeratin (AE1-3) expression is a robust marker to analyze the tumor cell fraction.

### The effects of cytotoxic treatment can be detected through cPARP staining and are reproducible and dose dependent

**Fig. 4:**
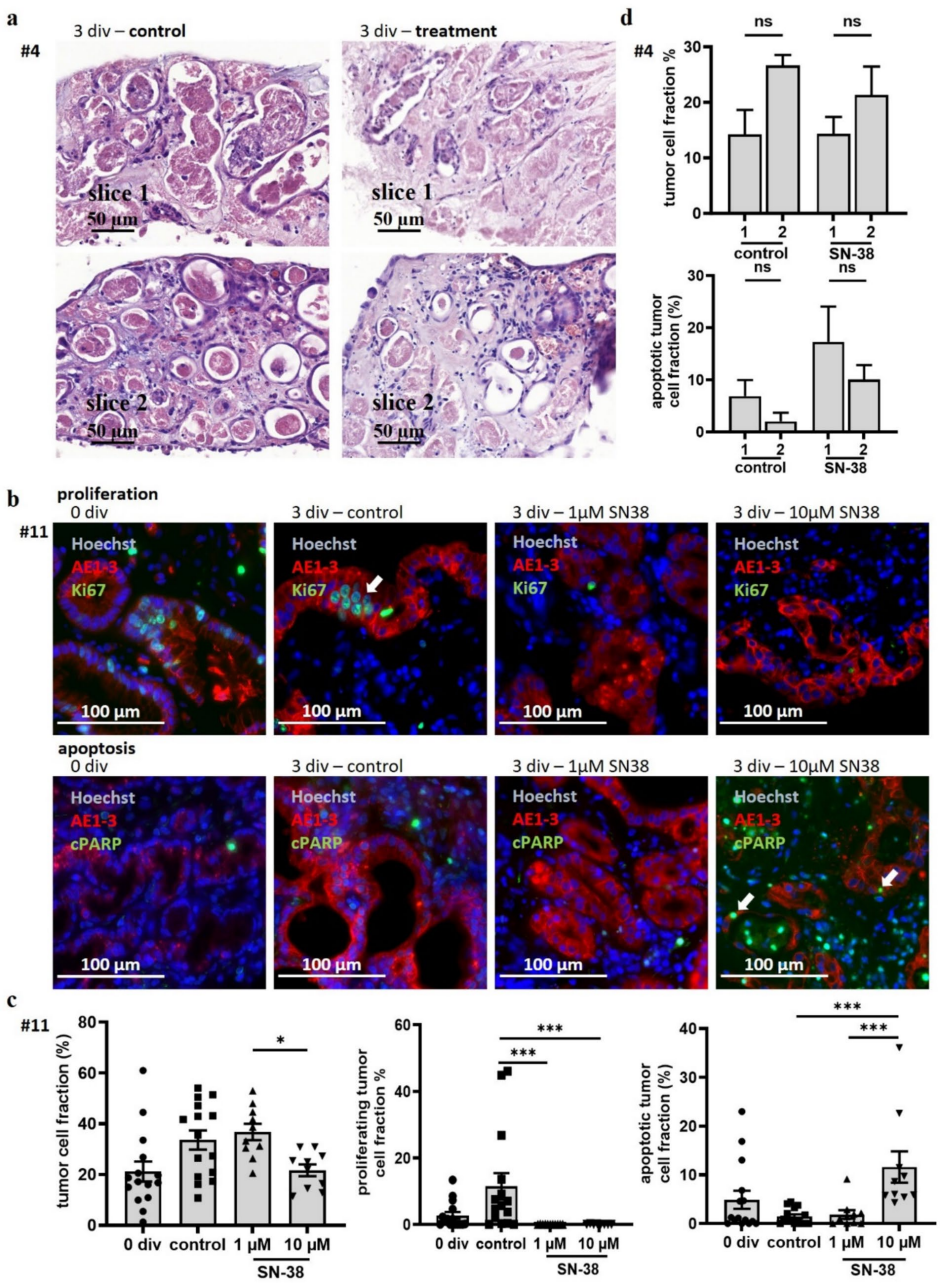
Assessment of quality characteristics of the culture method. (**a)** Slices from one endoscopic sample (#4) were put into duplets and were stained with H&E after 72 h (3 div) in culture each without treatment and with treatment. Each of the two slices shows similar morphological features. (**b)** Shown are slices from an esophagogastric adenocarcinoma at the time of obtaining (0 div) and after 72 h in culture (3 div), either not treated (control) or treated with 1 µM SN38 or 10 µM SN38. The slices were immunostained for pancytokeratin and ki67 or cPARP and counterstained with Hoechst. (**c)** For these slices, the tumor cell fraction (*p < 0.05, ordinary one-way ANOVA), proliferating tumor cell fraction (***p < 0.001, Mann‒Whitney test) and apoptotic tumor cell fraction (***p < 0.001, Mann‒Whitney test) were calculated. The mean with SEM is shown. (**d)** The slices from #4 (Fig. 4a) were analyzed regarding the tumor cell fraction and apoptotic tumor cell fraction. No difference was observed when comparing replicates

H&E staining revealed good cellular survival in untreated conditions, while treated conditions in some patients showed morphological features for apoptosis. We observed loose cell organization, small, fragmented nuclei, and cell detritus (Fig. 4a). To further analyze tumor survival and reproducibility, we conducted immunostaining for proliferation (Ki67) and apoptosis (cPARP, Fig. 4b). Comparing two single slices from tested duplets showed that tumor cells and apoptotic tumor cells did not differ significantly (Fig. 4c). Treatment with the active form of a second-line therapy, SN38, further showed a significant decline in the tumor cell fraction comparing 1 µM (36,71%) and 10 µM (21,64%). Whereas the proliferating tumor cell fraction was significantly (*p* ≤ 0,001) reduced in both treatment conditions, a dose-dependent rise in the apoptotic tumor cell fraction was observed (*p* ≤ 0,001; 1 µM, 1,87%; 10 µM, 11,6%) (Fig. 4d).

### Determining tissue response using the cytotoxic active compound of irinotecan

**Fig. 5:**
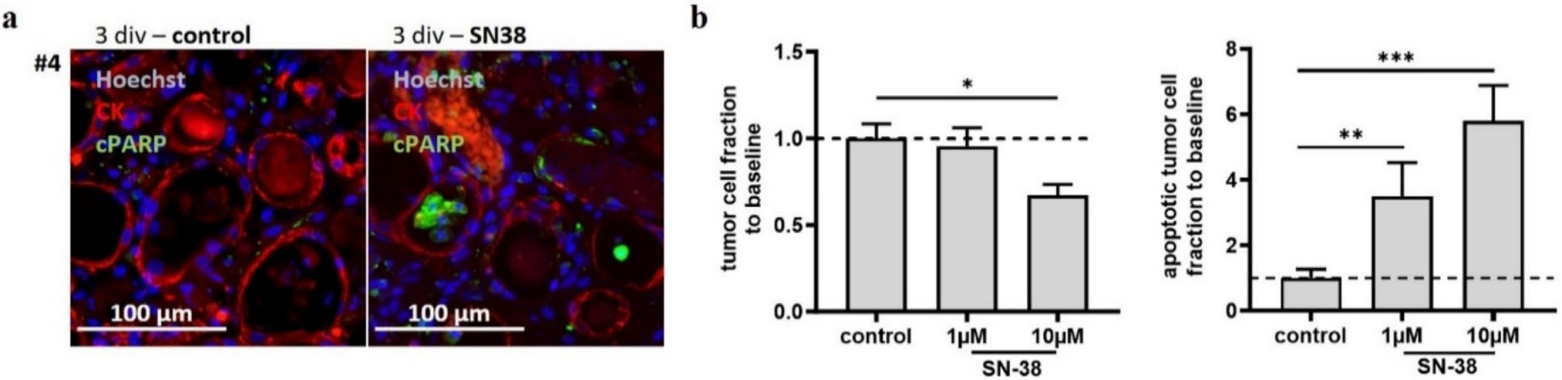
Determination of tumor cell response (**a)** #4 was treated with SN38, immunostained for pancytokeratin and cPARP and counterstained with Hoechst. (**b)** Shown are all cases that were treated with SN38 at two different concentrations (*n* = 3); the values are relative to the untreated control culture condition. The tumor cell fraction and apoptotic tumor cell fraction both showed dose-dependent effects. All cases in this study responded to SN38. (*p < 0.05, **p < 0.01, ****p < 0.0001, Mann‒Whitney test)

Determining the tumor responses to treatment was first investigated by utilizing the active compound of SN38. Due to its high cytotoxicity and its application as second-line therapy, most received biopsy samples were still not resistant and tumor cells should be susceptible. Treatment with SN38 showed a decline in tumor cell fraction and an increase in the apoptotic tumor cell fraction in all cases (Fig. 5b, n = 3). Experiments #3, #4 and #11 were treated with 1 µM and/or 10 µM SN38. For each condition, at least two to three tumor slices were analyzed. The difference in tumor cell fraction between the overall baseline and the normalized conditions that were treated with 10 µM SN38 was significant (*p* ≤ 0,05). The increase in the apoptotic tumor cell fraction was significant at both concentrations compared to the control condition (control vs. 1 µM, *p* ≤ 0,01; control vs. 10 µM, *p* ≤ 0,001). Dose dependency was detectable in all experiments, considering the two parameters of tumor fraction and tumor apoptosis (Fig. 5a, b).

### Determining individual susceptibility to 5-FU, FLOT and PD-1 inhibition

**Fig. 6:**
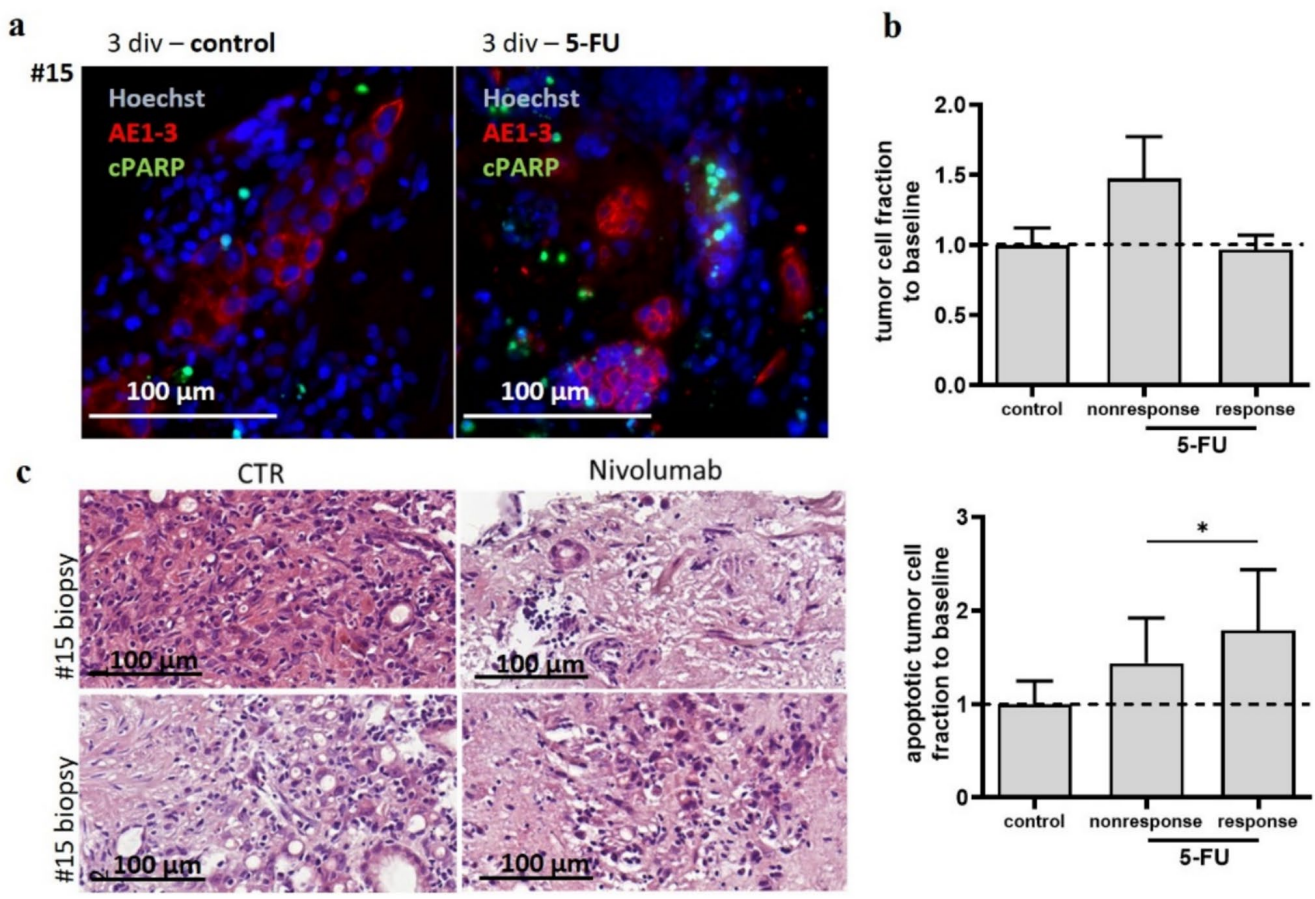
Effects of single 5-FU and PD-1 inhibition. (**a)** #7 was treated with 5-FU and immunostained for pancytokeratin and cPARP and counterstained with Hoechst. (**b)** Shown are cases that were treated with 5-FU (*n* = 3). Response and non-response were differentiated in the individual cases by apoptotic tumor cell fraction and tumor cell fraction. (*p < 0.05). The mean with SEM is shown. (**c)** Two biopsies of the same tumor were treated in ePDTCs with nivolumab. Both tissue samples demonstrate independently enhanced cellular apoptosis, reduced tumor cells and tissue remodeling *ex vivo*

Not all patient samples responded to 5-FU (1 µM) treatment. Two responding tissue samples (#7, #9) and one non-responding tissue sample (#8) were analyzed (Fig. 6ab). In experiment #8, a non-significant alteration of the tumor cell fraction compared to the control conditions (149% 5-FU) and a significantly lower apoptotic tumor cell fraction compared to the responding tissues (65% 5-FU, *p* ≤ 0.05) was observed. Regimen application was tested with FLOT. Most patients received neoadjuvant treatment with FLOT and showed consequently no response to ex vivo treatment. One patient receiving no prior FLOT treatment showed response in ePDTCs ex vivo (suppl. Table 1). To further investigate ePDTCs upon its relevance for immune checkpoint inhibition, we investigated three ePDTCs with PD-1 inhibition by nivolumab. The tissue cultures displayed clear responses by examining only the H&E staining. Considering the impact of PD-1 inhibition on the TME, we could observe tissue response in one single case, demonstrating major tissue remodeling and diminished tumor cell fraction, as observed previously in resections of gastric and lung cancer (Fig. 6). Two further cases are shown in suppl. Figure 1. A later diagnosed ulcerous tissue did not show alterations upon PD-1 inhibition and a carcinoma of the EGJ demonstrated well maintained tumor cells and TME, although some tissue remodeling occurred.

### The addition of patient-derived human serum to the corresponding culture medium led to enhanced susceptibility and better morphological preservation of ePDTC

To investigate whether the culture media has an impact on ePDTC, which was not observed in PDTC [20], we added 2% autologous serum and reduced the amount of FCS. Three samples could be treated with patient-derived autologous samples and one was treated with FLOT.

**Fig. 7:**
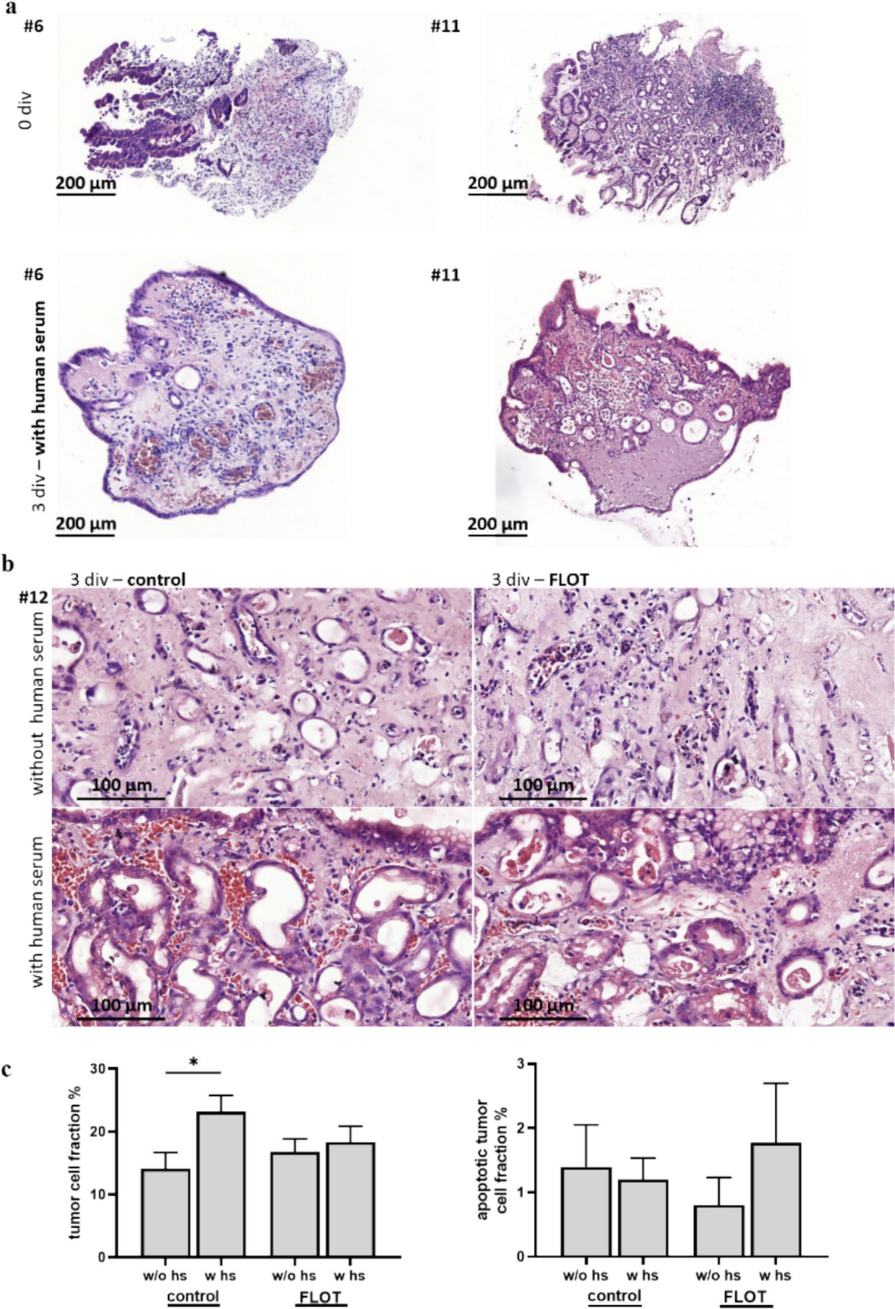
Effects of adding matching patient-derived serum to culture medium. (**a)** Two ePDTC are shown, which were cultivated for 3 days with human serum added to fetal calf serum. We observed that the tissue was surrounded by a newly formed epithelium when treated with human serum. (**b)** #12 was cultivated either with human serum added to fetal calf serum or only with fetal calf serum. H&E staining after 3 days for the control and FLOT conditions is shown. The conditions with human serum expose a greater quantity and quality of glands. (**c)** The control condition shows a significantly higher tumor cell fraction when cultivated with human serum (*p < 0.05, Mann‒Whitney test)

We observed better morphological preservation and in some ePDTCs a formation of an epithelial barrier in the presence of human serum in comparison with single FCS supplementation (Fig. 7a,b). The tumor cell fraction was significantly higher in the condition that contained human serum (Fig. 7c, + 9.8% abs., *p* ≤ 0.05), and although no difference of the tumor cell fraction was found under FLOT treatment, the apoptotic tumor cell fraction was altered (1.77% vs. 0.81%, *p* > 0.05).

## Discussion 

Endoscopic samples are crucial for tumor diagnosis and treatment assessment. As personalized medicine advances, we have adapted the PDTC technique specifically for endoscopic-derived PDTC. Personalized drug testing using this approach may help identify ineffective treatments, improving patient care and reducing healthcare burdens. Our results show that tissue from endoscopic biopsies can be successfully cultured, maintaining tissue integrity for up to four days ex vivo. Notably, time from diagnosis to treatment of about five weeks does not correlate with worse overall survival [21]. This time frame allows ePDTCs to serve as a practical model for evaluating drug combinations relevant to clinical settings or to determine adverse effects of treatment strategies. Although a direct correlation between ePDTC responses and patient outcomes has yet to be established, some correlations have already been observed [20, 22, 23]. However, an extended cohort of endoscopic samples is needed to validate clinical applicability. To account for tumor cell variability and tissue heterogeneity, we recommend obtaining at least four endoscopic samples per patient and distributing them across all test conditions. This approach offsets local tissue variability and potential sample loss, deviating from earlier methods that used random distribution of resected tissue [23].

To differentiate neoplastic from non-neoplastic tissue, we compared independent histopathological evaluations of tumor cell fractions, finding greater variability and standard error with manual histology, emphasizing the challenge of differentiating neoplastic tissue without specific immunohistochemical stains. Advanced techniques, such as multispectral imaging and high-resolution spatial transcriptomics, may improve the reliability of tissue response and biomarker identification, helping to track resistance mechanisms and evaluate immune therapies over time [24–29]. A primary benefit of ePDTC is the preservation of cellular complexity in its natural architecture. Studying cytotoxic agents, we observed cytotoxic effects not only on tumor cell but also on stromal cells. The high metabolic activity of tumor cells is addressed by cytotoxic agents but also cells of the stromal compartment respond [30]. Therefore, Fronik et al. developed an oxaliplatin prodrug, releasing L-buthionine-S,R-sulfoximine (BSO), an inhibitor of glutamate-cysteine ligase, the rate-limiting enzyme in glutathione biosynthesis and demonstrated reduced toxicity in an in vivo mouse model [31]. Although PDTCs of tumor resections will be more suitable, these complex model systems are suitable to define relevant improvements, enhancing the potency and reducing the burden of existing drugs for clinical use as they consider tissue penetration and the stromal compartment of the tumor. We used the active compound of irinotecan, an inhibitor of the topoisomerase I to induce dose-dependent cytotoxicity to define relevant read out parameters to differentiate tissue responses from non-responses. In the here investigated tumor entity, tumor fraction together with the apoptotic tumor fraction demonstrated applicability to evaluate tissue responses of cytotoxic agents. Our analysis showed that the applied low doses of irinotecan aligning with the clinical response rates of roughly 6% for irinotecan [32, 33]. Still, considering the bioavailability of irinotecan, the low dose still exceeds plasma concentrations [34]. Short-term cultivation, however, cannot only consider plasma concentrations but should also take tissue accumulation of the investigated compound into account. In our study, six of fifteen samples responded to standard treatments, whether this proof-of-concept investigation reflects clinical responses remains uncertain, but only larger cohort studies can confirm its effectivity. Prior studies support the utility of PDTCs derived from surgical specimens across various cancers [20, 24, 35, 36], though acquired resistance mechanisms, such as active drug efflux, reduced topoisomerase I expression, and enhanced DNA repair, could also influence results [32, 37].

The histological response post-chemotherapy is used to determine the tumor regression grade, a key prognostic factor [38]. As most collected samples, were collected post-chemotherapy with prior FLOT treatment, we could demonstrate tissue resistance in all cases with prior FLOT treatment. Single 5-FU treatment, however, was still effective in one PDTC sample analysis. Complete regression after perioperative FLOT therapy remains under 20% for locally advanced adenocarcinomas [7, 11, 39, 40]. Although leucovorin boosts 5-FU’s response rate from 20% to roughly 30% in colorectal cancers, single-agent 5-FU more effectively suppresses thymidylate synthase (TS) in human intestinal tissue [41], suggesting resistance mechanisms. As the folate metabolism is complex, it was suggested that gamma-glutamylhydrolase (GGH) expression may help to predict leucovorin efficacy in colorectal cancer patients [42–45]. Studies using patient-derived organoids identified the Krüppel-like factor-5 (KLF5) as a potential target to enhance oxaliplatin’s effectiveness, though this model may not capture total intratumoral heterogeneity [46], it demonstrates the relevance of these complex culture systems to define novel targets as resistance is mostly inevitable [46, 47]. KLF5 plays a role in stromal regulation of tumor progression via the CCL5/CCR5 axis, a tumor escape mechanism by instrumentalizing the immune cell compartment [48]. As the ePDTC system maintains intra-tumoral immune cells ex vivo*,* it is also feasible to determine tissue susceptibility to immune checkpoint inhibition. As the mode of action does not merely depend on metabolic activity, tissue re-organization needs to be carefully considered, determining ePDTC response. We show that histological analysis clearly reveals tissue susceptibility. Similar effects were observable in tumor resections; however, the tissue ex vivo might overestimate the susceptibility to immune therapy as the culture itself induces an activation of the immunological cell department [20, 24]. Addition of autologous PBMC to the media in a co-culture set-up might allow to mimic a systemic response to further address tissue response and also the induction of adverse events [18, 20]. The autoimmune reactions triggered by immune checkpoint blockade remain a significant challenge [18]. Establishing clinical correlations with immune checkpoint inhibition in ePDTCs could help identify biomarkers in ePDTC-PBMC co-cultures.

The use of patient-derived sera may enhance clinical correlation by better reflecting the tumor microenvironment. Autocrine–paracrine loops of growth factors in patient sera play a crucial role in activating signaling networks and survival pathways in cancer cells. The variability in growth factor levels underscores the importance of using whole patient sera for a balanced induction of signaling pathways rather than relying on artificially combined growth factors. Majumber et al. (2015) demonstrated that a 2% concentration of patient sera is sufficient to maintain native metabolic balance, while higher concentrations may interfere with key tumor survival pathways [49]. In this and other studies, autologous serum has been associated with improved growth rates, better tissue preservation, and modified treatment responses. However, one limitation is that the supplemented serum used throughout the culture period corresponds to the patient’s pre-chemotherapy serum, meaning it does not account for systemic treatment-induced changes. Additionally, the dynamic pulsatility of serum component release cannot be replicated in vitro. Since patient serum reflects individualized medication and infection statuses, its application should be selective for personalized drug testing. For clinical validation, it is essential to study ePDTC in parallel with patient treatment. Defining inclusion criteria and multicenter collaboration will further enhance generalizability to fully understand its clinical relevance.

Taken together, we refined the tissue slice culture protocol to develop ePDTCs from gastric and esophagogastric junction cancers. Testing therapeutic agents in a larger cohort will determine the effectiveness of ePDTCs for rapid, patient-specific treatment screening to overcome inefficient therapies and to save time for relevant treatment stratifications.

## Methods

### Specimens

Endoscopic samples from 31 patients were obtained for the establishment and investigation of clinical feasibility. Depending on the endoscopist's decision, one to seven biopsies were taken from different areas of the suspected tumor. In total, 14 cases of gastric cancer [*n* = 6] and esophagogastric junction cancer [*n* = 8] and one ulcer disease were included in this study and were taken by the Department of Endoscopy of the University of Leipzig Medical Center, Germany (Supp. Table 1). The others patient samples were excluded due to histological not proven adenocarcinoma (*n* = 3) or insufficient tissue (*n* = 7) quality or quantity (*n* = 6). This study was performed in line with the principles of the Declaration of Helsinki and was approved by the ethics committee of the Medical Faculty, University of Leipzig (AZ 370/13-ff). All patients provided their informed written consent to participate in this study.

### Ex vivo tissue slice culture

The samples were transported in media (Gibco, Life Technologies, Paisly, UK) supplemented with 1% penicillin/streptomycin (Gibco). The preparation was conducted immediately (*n* = 13) or after 24 h (*n* = 2) with adaptations as previously reported [31]. Briefly, endoscopic tumor specimens were cut into 350-µm thin slices using a tissue chopper (McIlwain TC 752; Campden Instruments, Lafayette, IL) and separated with forceps and a surgical knife by means of a binocular microscope. Up to three tissue slices were positioned on semiporous membrane inserts (Millipore Corporation, Billerica, MA) [58]. Six-well plates were prepared with 1 ml RPMI-1640 culture medium, 1% penicillin/streptomycin, 1% L-glutamine (Gibco), 1% amphotericin B (Carl Roth, Karlsruhe, Germany) and either 10% fetal calf serum (*n* = 12; fetal calf serum [FCS]; Invitrogen, Darmstadt, Germany) or 8% FCS and 2% autologous serum (*n* = 3). The inserts were placed onto the medium-filled wells, and the plates were cultured for 48 h (*n* = 2) or 72 h (*n* = 13) at 37 °C in humidified air with 5% CO_2_. The medium was exchanged every other day.

### Experimental set-up

Each endoscopic specimen pictures different parts of the tumor. Different arrangements to obtain reliable and reproducible results were investigated. Slices obtained from specimens including more than one sample (*n* = 7) were either pooled and randomly distributed (*n* = 3) or assorted by condition (*n* = 4). The tissue slices were treated for 48 h (*n* = 11) or 72 h (*n* = 1) with either cisplatin (10 µM, *n* = 5) or SN38, the bioavailable component of irinotecan, (1 µM, *n* = 2; 10 µM, *n* = 2), or 5-FU (10 µM, n = 3) or FLOT (5-FU: 10 µM, folinic acid: 10 µM, oxaliplatin: 20 µM, docetaxel: 0.1 µM, *n* = 4). FLOT was applied for 2 h, followed by 5-FU for 46 h. The substances were diluted with culture medium, and the control groups were treated with culture medium without cytotoxic supplements. The medium was changed every 24 h.

### Staining

Slices were fixed with paraformaldehyde (4%) overnight, embedded in paraffin and cut into 5-µm sections. Hematoxylin and eosin staining was performed to assess tissue quality and tumor areas. Immunofluorescence staining was performed to evaluate the percentage of apoptotic and proliferating tumor cells. Therefore, sections were deparaffinized, and antigen retrieval was achieved by incubation in citrate buffer for 2 × 20 min at 95 °C. Sections were washed and blocked with 5% normal goat serum (Jackson ImmunoResearch, Suffolk, UK) for 30 min. Sections were incubated overnight with primary antibodies diluted in 0.5% bovine serum albumin against either cleaved PARP-1 (cPARP, Abcam, Cambridge, UK, ab32064, rabbit monoclonal, 1:100) and cytokeratins (Anti-Cytokeratin Cocktail, Clone AE1 & AE3, BioGenex, California, USA, MU071-UC, mouse, 1:100) or against Ki67 (DCS, Hamburg, Germany, KI681 C01, rabbit, 1:200) and cytokeratins. Sections were washed and labeled with secondary antibodies (568 goat-anti-mouse, 647 goat-anti-rabbit, Alexa Fluor, Invitrogen, Eugene, Oregon, USA). Nuclei were stained with Hoechst 33,342 (Sigma-Aldrich, St. Louis, Missouri, USA).

### Analysis/response evaluation

H&E sections were analyzed using slide scans (Pannoramic SCAN and CaseViewer, 3DHistech, Budapest, Hungary). Immunofluorescent pictures were taken by an Olympus BX51 Fluorescence Microscope. From every section, two to five positions were recorded. Pictures were analyzed automatically using ImageJ [59] with a sequence of image processing and pixel counting algorithms, which was previously published by our group [31]. Briefly, a cytokine-positive area was created to match the positive nuclei staining of Hoechst and cPARP. Pixels that were then double-positive for Hoechst and cytokeratin were counted as total tumor cells. Pixels that were additionally positive for cPARP or Ki67 were counted as triple-positive tumor cells. The percentages of the apoptotic or proliferating tumor cell pixel to the total tumor cell pixel were referred to as the apoptotic or proliferating tumor cell fraction. Mean slice values were calculated and used to calculate mean values for each condition.

### Statistical analysis

For the statistical analysis and visualization, we employed GraphPad Prism 9 version 9.5.0 for Windows, GraphPad Software, San Diego, California USA, www.graphpad.com. Each experiment was tested for a normal distribution of the measured results using the Shapiro‒Wilk test. If a normal distribution could be assumed, we utilized one-way ANOVA with Bonferroni's multiple comparisons test to determine the significance level. In cases where a normal distribution could not be assumed, we employed the Mann‒Whitney test. A significance level of P < 0.05 was considered statistically significant.

## Supplementary Information

Below is the link to the electronic supplementary material.

Supplementary file 1 (DOCX 1600 kb)

## Data Availability

The datasets generated during and analyzed during the current study are available from the corresponding author upon reasonable request.
