# Supplementary material for: Personalized drug stratification using endoscopic samples to assess ex vivo gastric cancer tissue susceptibility to chemotherapy and immune checkpoint inhibitors
Source: Clin Exp Med. 2025 Jun 4;25(1):188. doi: 10.1007/s10238-025-01694-z (PMC12137430; doi:10.1007/s10238-025-01694-z)
Supplement: Supplementary file 1 — (DOCX 1600 kb) [file 10238_2025_1694_MOESM1_ESM.docx]

# Supplement

## Supplementary Figures


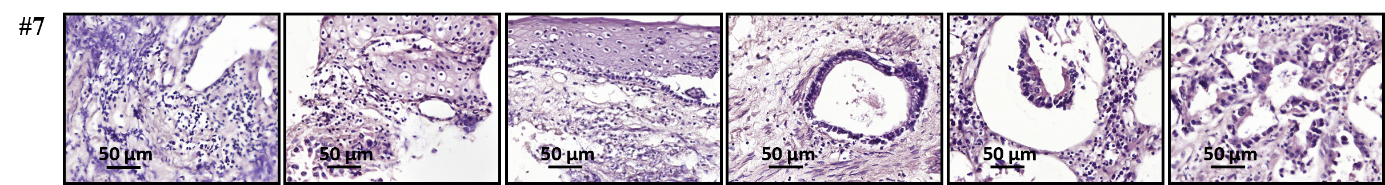


**Supp. Fig. 1: Six slices of** one biopsy from a patient diagnosed with esophagogastric junction cancer. There are parts of the squamous epithelium and parts of the mucosa.


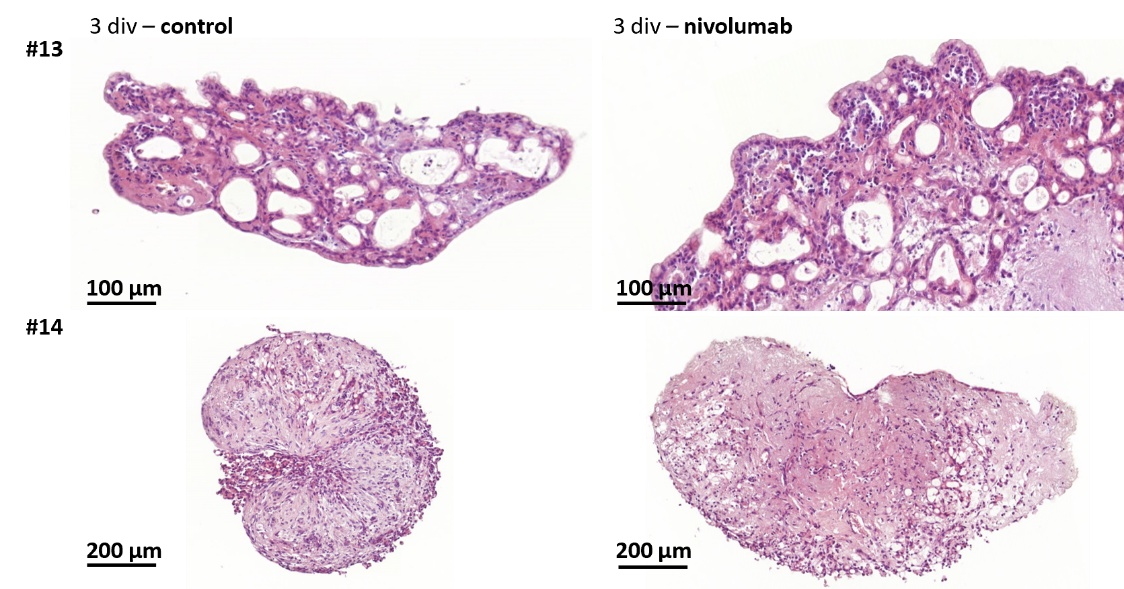


## Supp. Fig 2: Different response to nivolumab in two cases of gastric cancer (#13, #14). #13 shows no response to the treatment, with no apoptotic cell bodies observed. The tumor cells continue to proliferate, and the tumor maintains its characteristic adenomatous structure. #14 demonstrates a partial response, with an increase in apoptotic cell bodies and a reduction in stromal tumor cell proliferation.Supp. Table

| **#** | **Entity** | **Stage** | **Histology** | **Treatment before biopsy** | **Treatment after biopsy** | **Latest information** | **Treatment in culture** | **Response seen in culture** |
| --- | --- | --- | --- | --- | --- | --- | --- | --- |
| **1** | gastric cancer | advanced | Diffuse, G3 | None | n.a. | 12.02.2016: patient died | Cisplatin | Non-Response |
| **2** | gastric cancer | advanced | Diffuse, signet ring cell. G3 | 7/2015 6x FLOT  1/2016 Oxaliplatin | n.a. | 24.04.2016: patient died | Cisplatin | Non-Response |
| **3** | EGJ | Locally advanced | Diffuse, signet ring cell | 4 cycles FLO | n.a. | 16.08.2018: spinal and lymph node metastases | SN-38 | Response |
| **4 (9)** | EGJ | Locally advanced | Barrett's carcinoma | 4 cycles FLOT | n.a. | 16.12.2020:  no indication of recurrence | SN-38 | Response |
| **5 (11)** | EGJ | locally advanced | Diffuse, G3  (MSI-negative) | None | 4 cycles FLOT | 21.08.2019: Palliative discharge for home end-of-life care | Cisplatin | Non-Response |
| **6 (13)** | EGJ | Early local | Barrett's carcinoma | None | n.a.  (Emergency esophageal resection) | 03.08.2017: Patient died from postsurgery complications | Cisplatin | Non-Response |
| **7(21)** | EGJ | advanced | Intestinal, G2 | None | Palliative first-line mFOLFOX | 18.07.2019: Discharge to nursing home in a palliative setting | Cisplatin  5-FU | 5-FU  Non-Responder |
| **8(23)** | EGJ | Locally advanced | Intestinal, G2 | None | 4 cycles FLOT | 09/2022: no indication of recurrence | 5-FU  FLOT | 5-FU Non-Responder  FLOT Non-Responder |
| **9 (24)** | Gastric cancer | advanced | Diffuse, G3, signet ring cells | None | 6 cycles FLOT | 01.04.2019: patient died | 5-FU  FLOT | 5-FU Responder  FLOT Non-responder |
| **10 (25)** | Gastric cancer | Locally advanced | n.a. | None | Capecitabine palliative | 29.07.2020: patient died | FLOT | Response |
| **11(26)** | EGJ | Locally advanced | Diffuse, G3 | 4 cycles FLOT | 2 cycles FLOT | no indication of recurrence until 0ct. 2022 | SN-38 | Response |
| **12(28)** | Gastric cancer | advanced | Diffuse, signet ring cell, G3 | None | n.a. | 17.01.2019: Handover for treatment initiation at local oncology with FLOT | FLOT | Non-Response |
| **13(29)** | Ulcus |  |  |  |  |  | Nivolumab | No effect |
| **14(30)** | Gastric cancer | advanced | Intestinal, signet ring cell, G3 | For lung cancer: cisplatin, etoposide | None, palliative care | unknown | nivolumab | Partial response |
| **15 (31)** | Gastric cancer | advanced | Diffuse, Signet ring cell, G3 | None | Nivolumab, FLO | 26.08.2022: patient died | nivolumab | Response |

**Supp. Table 1:** Patient and experimental data. Abbreviations: n.a.=not applicable; FLOT=fluorouracil, leucovorin, oxaliplatin, docetaxel; FLO= fluorouracil, leucovorin, oxaliplatin; EGJ=esophagogastric junction cancer; SN-38=active compound of irinotecan; MSI=microsatellite instability; mFOLFOX=modified regimen of folinic acid, fluorouracil, oxaliplatin; 5-FU=5-fluorouracil.
